# Which Compounds Contribute Most to Elevated Soil Pollution and the Corresponding Health Risks in Floodplains in the Headwater Areas of the Central European Watershed?

**DOI:** 10.3390/ijerph15061146

**Published:** 2018-06-01

**Authors:** Jan Skála, Radim Vácha, Pavel Čupr

**Affiliations:** 1 Research Institute for Soil and Water Conservation, Žabovřeská 250, 15627 Prague, Czech Republic; vacha.radim@vumop.cz; 2 Research Centre for Toxic Compounds in the Environment, Masaryk University, Kamenice 753-5, 62500 Brno, Czech Republic

**Keywords:** soil pollution, floodplain, human health risk, compositional data

## Abstract

The main topic of this study is a human health risk assessment of a defined exposure scenario in the floodplain soils of the headwater areas of the central European watershed, with the aim of exploring both multivariate and regional data structures. Flood-prone areas are recognized worldwide to be susceptible to contamination and its redistribution. Contributions of various classes of toxic compounds (organochlorine pesticides (OCPs), polycyclic aromatic hydrocarbons (PAHs), polychlorinated biphenyls (PCBs)) to human health risks were assessed in a screening risk assessment. However, due to the relative nature of our data and a high PAH dominancy over the data ensemble, reliance solely on the standard statistical processing of raw data might lead to incomplete insight into the structure of the multivariate data. Explanatory analysis of the data structure using the compositional approach was found to be beneficial to elucidating human health risk profiles and provided robust evidence that a contrast between agricultural and airborne industrial pollution controlled the whole human toxicological variation of persistent organic pollutants (POPs) in floodplain soils. These results were effectively quantified with the subcomposition of benzo(*a*)pyrene, DDT, and alpha-hexachlorocyclohexane (aHCH), allowing for an interpretation of structural differences in regional pollution patterns, which conferred different extents and compositions of human health risks in floodplain soils.

## 1. Introduction

The contamination of the environment by hazardous substances such as persistent organic pollutants (POPs) is a worldwide public health concern. POPs show serious toxic effects on humans as well as wildlife in very low concentrations [1]. One of the matrixes that acts as an effective sink of these toxic chemicals is soil [1,2]. Whilst floodplain soils are renowned for their fertility, attributed to nutrient inputs, the same enrichment process renders these soils susceptible to contamination by various pollutants [3]. They are subject to further redistribution and transformation processes, which are a matter of great importance in floodplain soils [4]. Although the rivers in the Czech Republic underwent significant changes in the quality of surface waters during the 1990s due to a decline in industrial and agricultural activity associated with political changes [5], increased concentrations of various pollutants in alluvial soils in Central Europe can still be found [6,7,8]. The Czech Republic, as a typical Central European country, is located in the European Watershed area; hence, several European great rivers have their headwaters located there. Since ongoing climatic changes may increase the risk of extensive floods in Europe [9], both the health and environmental consequences of the old burdens as well as of emerging pollution should be monitored and controlled in the floodplains. Humans are a sensitive target of POP bioaccumulation and its associated adverse effects [10]. Also, potential transfer to the human body may occur through different pathways of direct exposure [11,12,13] or dietary exposure [14].

Since our knowledge of POP pollution profiles in floodplain soils in the Czech Republic is still fragmentary and there are many data gaps regarding nondietary exposure pathways, extensive soil sampling combined with a screening assessment of human health risks was conducted. To assess the health risks connected to the presence of POPs in floodplain soils, we proceeded as follows. In the first step, a countrywide inventory of floodplain soils was performed with a focus on the properties and contamination levels of cultivated soils. As a second step, an established model [15] was adapted to field data for evaluation and interpretation of exposure to toxicologically important organic chemicals. Contributions of various classes of toxic compounds (PCBs, organochlorine pesticides (OCPs), PAHs) to total human health risks were then assessed using the main principles of statistical analysis for compositional data in this study.

## 2. Materials and Methods

### 2.1. Sampling and Data Acquisition

The target areas for soil sampling were selected using a geographic information system (GIS)-based approach, bringing together nationwide digital data on soil distribution, land use, and hydrological floods in the Czech Republic. The soils were preferentially sampled in those areas vulnerable to inundation (the five-year floodplains) where the risk of extensive inundations met a high cropland extent. For each sampling site (*n* = 100), a mixed sample consisting of 10 individual probes with depths of 0–30 cm was collected between 2010 and 2015. The basic soil properties (e.g., total organic carbon, soil texture characteristics) were also determined.

### 2.2. Laboratory Analysis

Chemical measurements for POPs (PAHs, PCBs, OCPs) were performed in the accredited laboratory of in the Trace Analysis Laboratories in the Research Center for Toxic Compounds in the Environment. All soil samples (10 g) were spiked with a 50 µL solution of perdeuterated PAHs and extracted using automated Soxhlet extraction for 40 min with dichloromethane in a Büchi extraction unit (Büchi, Uster, Switzerland). The extract was divided into a ratio of 1:9 for PAHs and PCBs + OCPs. For PAH analysis, the volume was reduced under a gentle nitrogen stream at ambient temperature and further covered by silica purified using Soxhlet for 8 h with dichloromethane. The fractionation achieved on a silica gel column was then used for the analysis of the PAH content. The sample was eluted using a mixture of dichloromethane and hexane, and spiked with deuterated p-terphenyl (50 µL, 4 µg/mL) surrogate standard. PAH contents were quantified using gas chromatography–mass spectrometry (GC–MS) (Agilent GC 7890/MS-MS Triple Quadrupole 7000B-Agilent, Santa Clara, CA, USA) equipped with a 60 m × 0.25 mm × 0.25 µm DB5-MS capillary column (J&W Scientific, Folsom, CA, USA). The isotope dilution method was used for quantification of the compounds. Helium was used as an inert carrier gas. For PCBs and OCPs, the volume was reduced under a gentle nitrogen stream at ambient temperature, covered by purified silica, and transferred into a vial. Finally, PCB 121-81 (0.2 µg/mL) was added as an internal standard. The extract was cleaned on an H_2_SO_4_-modified (44% *w*/*w*) silica column, and analytes were eluted with a 20 mL DCM/*n*-hexane mixture (1:1, *v*/*v*). Samples were quantified using gas chromatography–mass spectrometry (GC–MS) (Agilent GC 7890/MS-MS Triple Quadrupole 7000B) equipped with a 60 m × 0.25 mm × 0.25 µm HT8 SGE column (SGE Analytical Science, Ringwood, Australia) and with helium as a mobile phase. The isotope dilution method was used for quantification of the compounds. Certified reference materials (Cambridge Isotope Labs soil standard RM-0002) and laboratory blanks were analyzed with each set of POP samples.

### 2.3. Human Health Risk Assessment

In this study, a screening risk assessment was performed for the estimation of the human intake of soil contaminants and consequent risks as suggested by the United States Environmental Protection Agency [16]. The risks were quantified under the present environmental conditions for the selected exposure scenario for those compounds that may pose the most significant potential risk to humans. The selected human exposure pathways addressed in this study included the inhalation of particles, dermal contact, and ingestion. Risk characterization was considered separately for carcinogenic and noncarcinogenic effects.

From a numerical perspective, the potential human health risks were estimated by a confrontation of the actual environmental concentration (PEC) with the calculated soil screening level (SSL) for human health risks of soil contamination (see Equations (1) and (2)). The SSLs represented the risk-based soil concentrations determined for the involved chemicals from equations combining exposure assumptions with common toxicity criteria. The SSLs based on noncarcinogenic risks were estimated using the site-specific exposure parameters (in Table 1) substituted into Equation (1).

Human exposure risks were assessed for certain exposure scenarios. Setting the exposure parameters specified the extent to which the model accurately represented reality. The site-specific exposure parameters were set out according to typical conditions of these soils in alluvial areas (Table 2 and Table 3). As such, they are a fair reflection of the exposure of farmers, although other population groups, such as residents and bystanders, may also be exposed to these compounds due to soil pollution. The detailed methodology is also described in Čupr et al. [15], whose paper includes a discussion of the factors that may result in either an overestimation or an underestimation of the risks.

(1) $$SSL_{i}=\frac{THQ\cdot BW_{c}\cdot AT_{c}}{EF_{r}\cdot ED_{c}\left[\left(\frac{1}{RfD_{o}}\cdot \frac{IRS_{c}}{10^{6}mg/kg}\right)+\left(\frac{1}{RfD_{o}}\cdot \frac{SA_{c}\cdot AF_{c}\cdot ABS}{10^{6}mg/kg}\right)+\left(\frac{1}{RfD_{i}}\cdot \frac{IRA_{c}}{VF_{s}\;or\;PEF}\right)\right]}$$

The SSLs based on carcinogenic risks were estimated using the site-specific exposure parameters (in Table 2) substituted into Equation (2). The results were compared with the carcinogenic benchmark level, i.e., exposure posing an upper-bound lifetime excess cancer risk of 1 × 10^−6^ (one cancer occurrence in one million people).

(2) $$SSL_{i}=\frac{TR\cdot AT_{c}}{EF_{r}\left[\left(\frac{IFS_{adj}\cdot CSF_{o}}{10^{6}mg/kg}\right)+\left(\frac{SFS_{adj}\cdot ABS\cdot CSF_{o}}{10^{6}mg/kg}\right)+\left(\frac{InhF_{adj}\cdot CSF_{i}}{VF_{s}\;or\;PEF}\right)\right]}$$

The hazard quotient assumes that there is a level of exposure below which it is unlikely for even sensitive populations to expect any adverse health effects. In our case of exposure to multiple chemicals, a final cumulative health risk (hazard index for *n* pollutants) related to each sampling site was calculated as a sum of the partial quotients for the *i* involved compounds in Equation (3). A final HI < 1 indicates that no adverse health effects are likely to occur at the present exposure dose, and the human health risks (HHRs) are currently acceptable.

(3) $$HQ_{HUMAN}=PEC_{i}/SSL_{i}$$

(4) $$HI_{HUMAN}=\sum_{i=1}^{n}HQ_{HUMAN}$$

In the last step we calculated the relative contribution of each substance to an overall hazard index (HI_HUMAN_):

(5) $$p_{i_{RISK_{HUMAN}}}=HI_{HUMAN_{i}}/HQ_{HUMAN}.$$

### 2.4. Data Manipulation and Statistical Analysis

Principal component analysis (PCA) and cluster analysis were performed to evaluate the compositional similarities among samples. PCA is a widely used explanatory technique in terms of variance explanation and dimension reduction. The goal of any cluster analysis is to recognize homogenous clusters with the assumption that an underlying group structure exists. Following the expository potential of both tools, these were applied to explore the compositional variability of major components in our health risk survey. Nevertheless, these techniques cannot be directly applied to compositional data (objects described by vectors comprising parts of some whole) because they do not agree with the geometrical structure of the feature space [17]. Since the benefits of using the ratios between components have been recognized in statistical theory [18], the log-ratio transformations were proposed to overcome the shortcomings of analyzing compositional data. The advantages of both the centered log-ratio (clr) transformation [18] and the isometric log-ratio (ilr) transformation [19] may be exploited in the framework of a robust PCA [20]. A matrix of relative contributions of each pollutant to total HI_HUMAN_ for each locality was prepared before the statistical analysis (Equation (5)). In order to avoid misinterpretation of the results, half of the detection limit was used in all summations and for statistical analyses. However, a sizeable proportion of all data with an identical value can influence the multivariate analysis of compositional data [21]. This may be the case of some PCBs and hexachlorocyclohexane isomers (HCHs), where congeners PCB 52 and PCB 118 as well as the bHCH isomer proved to have especially significant proportions of observations below the detection limit (see Table 3). This effect of abundant identical values may be relieved by using the relative contributions of each compound to calculate the human risks from our point of view. Hence, all of the compounds were included in the multivariate analysis. PCA was performed after the matrix had been transformed using the ilr transformation and the resulting loadings and scores were back-transformed to the clr-space—for details, see Filzmoser et al. [20]. For clustering purposes, the fuzzy *c*-means algorithm (FCM) was employed to provide a degree of membership to each one of the resulting clusters. The fuzzy clustering problem can be characterized as classifying a given set of objects to fuzzy subsets, each of which is represented by its prototype with the most typical group characteristics [22]. Palarea-Albaladejo et al. [23] and Templ et al. [21] empirically tested various clustering algorithms on compositional datasets adopting a log-ratio approach. Following their experience, the clustering results were obtained using the FCM on ilr-transformed data. In the last steps, some graphical tools were used to pool the multivariate results. Therefore, a biplot [24] was used to graphically represent the variability in the entire composition and to assist the selection of relevant subcompositions that retain as much of the total variability in the entire composition as possible. The exact procedure for constructing the biplot adapted to compositions was presented in Filzmoser et al. [20]. For its statistical interpretation, see Aitchison and Greenace [25]. Finally, PCA results led to a ternary diagram, enabling readers to obtain lower-dimensional insights into the nature of the compositional dataset. These plots were subsequently used to represent clusters using the well-suited subcomposition defined according to the PCA results. The compositional biplots constructed on the basis of PCA and ternary plots have been shown to be powerful exploratory tools for various compositions [20,25]. Following this, we used them for visualization and deeper understanding of the survey results.

Since we analyzed only some of the possible compounds in the soil sample, our chemical dataset actually represents a subcomposition. Moreover, we replaced some measured values (mass ratios, μg/kg) by some computed ones (health risk estimations combining exposure assumptions with toxicity criteria) in our analysis. When applying any statistical method to compositions, three conditions should be fulfilled: scale invariance, permutation invariance, and subcompositional coherence [18]. Thus, using a log-ratio approach was an essential step to avoid the pitfalls of compositional data.

All of the computations and visualizations were conducted with the “robCompositions” [26], “compositions” [27], “ape” [28], and “cluster” [29] packages for the R statistical software (R CORE TEAM, Vienna, Austria). The regional patterns of the multivariate analyses results were spatially visualized using ArcGIS 10.2 (ESRI, Redlands, CA, USA).

## 3. Results

When our findings were compared to concentrations of POPs in Czech agricultural soils reported by Holoubek et al. [1], a similar trend could be observed for all POP compounds. Median concentrations were lower in our dataset (PAHs, DDTs, HCHs, PCBs, hexachlorobenzene (HCB); see Table 3), but usually with a wider range of values (PAHs, DDTs, HCHs). The results revealed a general high variability of POP concentrations in floodplain areas. In a previous study, both the magnitude of the estimated human health risks and the magnitude of hazards quotients of involved POPs were surveyed. Moreover, the highest estimated human health risks were found only in floodplain areas with high contents of PAHs exceeding the Czech legislation limits for agricultural soils [30]. In a closed system of compositional data (summing up to a constant), there is little space for all other compositional parts to vary in the case of exceptionally high relative contributions of carcinogenic PAHs (c-PAHs) to the estimation of HHRs (i.e., benzo(*a*)pyrene > benzo(*b*)fluoranthene > benzo(*a*)anthracene in Table 3). These proportional effects may be relieved by applying a log-ratio approach to the compositional dataset [31]. This approach allows researchers to explore those variable relationships that may be forced by the overall predominance of PAHs in health risk estimations and hence allows an interpretation of both known and unexpected patterns in the opened data.

### Multivariate Results

**Figure 1:**
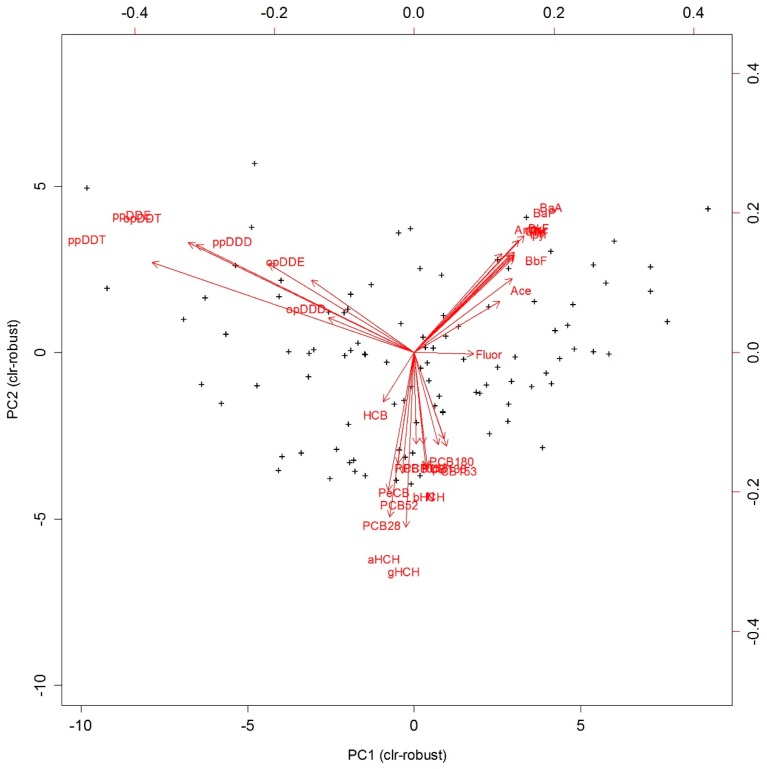
Biplot of the first two robust principal components (PCs) for the centered log-ratio (clr)-transformed major components (explained variation: PC1, 51%; PC2, 20%).

Figure 1 summarizes the compositional biplot obtained with the robust PCA using the log-ratio approach and allows the interpretation of compositional variability. The origin of the compositional biplot represents the center of the compositional dataset; links between ray vertices represent the variance of the log-ratios between two components; and rays represent the clr-transformed variables. If several rays are collinear, the relative variability within the subcomposition formed by these variables might be one-dimensional and might represent a process influencing these components in a similar way [31]. If ray vertices coincide, then the two involved compositional parts may be assumed to be redundant. Nevertheless, when interpreting rays, one has to consider their dependency on the center as they represent clr-transformed variables. Hence, the constellation of links is fundamental when interpreting the compositional covariance structure in these biplots. Short links indicate variables that are highly proportional. Orthogonal links suggest two involved subcompositions to be uncorrelated.

By applying these principles, we can clearly see several coincident rays consistently pointing towards diverging directions and connected with relative short links indicating three distinct groups of variables in Figure 1:

1 PAH compounds,
2 DDTs and metabolites, and
3 PCBs, HCHs, and PeCB.

**Figure 2:**
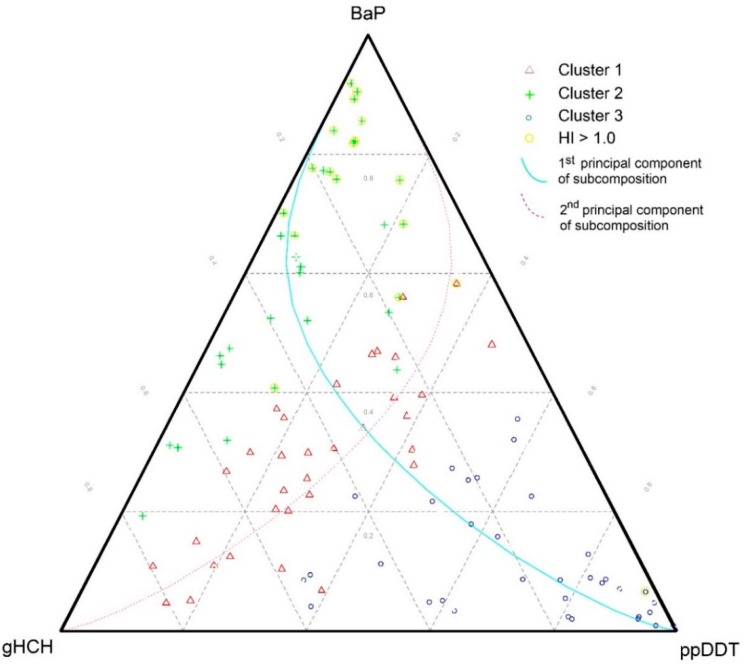
Ternary diagrams of the subcomposition (benzo(a)pyrene (BaP), gamma-HCH (gHCH), ppDDT) with distinguished three-group fuzzy partitions and the first two principal components (PCs) of the presented subcomposition (note that the samples with HI_HUMAN_ > 1.0 are highlighted with a yellow circle).

**Figure 3:**
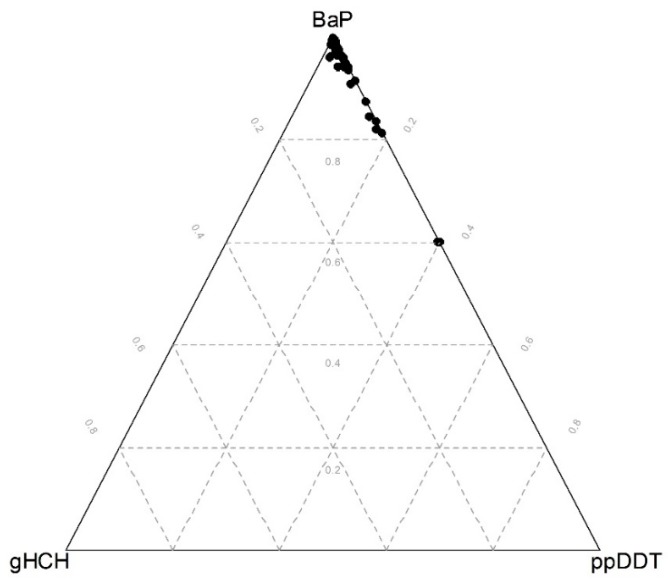
Ternary diagrams of the subcomposition (BaP, gHCH, ppDDT) using raw data components.

Short links between those compounds in each group indicate variables that are highly proportional and predetermine redundant variables. On the other hand, the orthogonality of the three groups suggests some uncorrelated log-ratios (for instance, PAHs/DDTs vs DDTs/HCHs). This may be useful in the investigation of subcompositions for possible independence and the selection of variables to be visualized in ternary plots. Since such clear patterns were found in the preceding descriptive analysis, a well-suited ternary diagram of the subcomposition formed by the distinctive rays from each group could record the whole variability exceptionally well [31]. This is the case for the subcomposition (BaP, gHCH, ppDDT) in Figure 2 and Figure 3. Since the proportion of explained variance captured by the two first principal components (PCs) reached 71%, the PCA results as well as the derived ternary diagrams may be considered a good approximation to the real structures in the data set. The robust PC1 can be seen as a contrast between the components of the PAH group and DDT group, while PC2 is dominated by elements of the third group in both Figure 1 and Figure 2. The analysis of the compositional biplot suggests that most of the variability was controlled by two main factors—PAH contamination connected to higher atmospheric pollution inputs in the catchment, and DDT contamination connected to the agricultural intensity.

**Figure 4:**
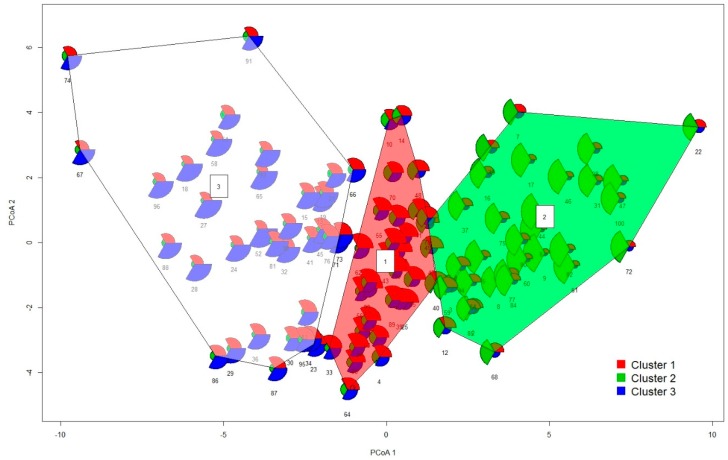
Membership probabilities and assigned cluster number in a graphical representation of principal coordinate analysis (PCoA) on Aitchison distances between sampling localities.

**Figure 5:**
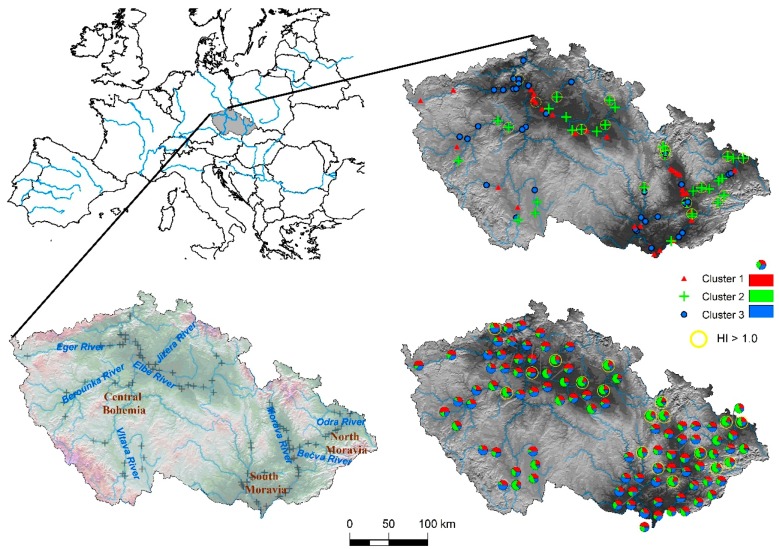
Membership probabilities and assigned cluster number in a geographical representation for regional interpretation (note that the samples with HI_HUMAN_ > 1.0 are highlighted with a yellow circle).

A three-group solution was considered as the optimal grouping of the dataset in the cluster analysis. The membership probabilities from the FCM approach are graphically shown for each sample in Figure 4 and Figure 5. Comparing clustering and ordination results can be beneficial to explaining the differences between groups of sites. Compositional biplots summarize the variance relationships within the compositions; nevertheless, they are chosen to maximize the variance of the projected data cloud and are not tailored to highlight between-group differences [31]. For this purpose, a graphical representation of principal coordinate analysis (PCoA) was used on the Aitchison distance matrix, in which the eigenvectors are scaled to the square root of the corresponding eigenvalues (Figure 4). Gower [32] showed that eigenvectors scaled in this way preserve the original distance among the objects in the distance matrix. We can see that the clusters are basically defined by their projections along the first two principal components, according to their human risk patterns and the differences in relative contributions of DDTs, HCHs, and PAHs to the calculated human risks. Hence, the three-group clustering corresponded well to the variation within the composition.

In fact, if we used a ternary plot (Figure 2) to represent the established subcomposition (designed by representative compositional parts formed by some of the longest rays from each group in the compositional biplot in Figure 1), the clusters are clearly distinguished. The data as much as the grid lines were centered and the first two principal components of the subcomposition were added to the ternary plot. The data were centered by calculating the closed geometric mean, which was obtained as the closure of the vector of geometric means of each component. In this way, the geometric center of the observed data could be moved to the barycenter of the simplex space, allowing us to better visualize the structure of the sample [33]. When comparing the ternary diagram obtained for raw data (Figure 3) with that obtained for the centered log-ratio-transformed data (Figure 2), we could see that the raw data tended to collapse on a vertex, obscuring the structure (BaP in our case). Hence, only limited information can be extracted from raw proportions due to the proportionality effect [31]. Comparing both diagrams, the actual benefits of the log-ratio approach can be seen. Note that the positions of the clustering groups within the subcompositional representation using ternary diagrams (Figure 2) agree with the partition results of fuzzy clustering in Figure 4. It can be again seen that Clusters 1 and 3 are the groups with the most similar characteristics, and also that Cluster 2 is the most different group. This may be explained by the fact that Cluster 1 and 3 are both dominated by relatively high contributions of various OCP compounds and thus may be both influenced to some extent by long-term intensive husbandry. Finally, the processes behind the clusters were investigated according to the relative weight of each POP component in every group using centered data, according to Palarea-Albaladejo et al. [23] (Figure S1).

## 4. Discussion

Cluster 1 included samples with a higher proportion of organochlorine pesticides with prevailing agricultural origin (HCHs, PeCB, HCB) together with samples with higher organochlorine compounds of industrial origin (PCBs). On the contrary, Cluster 2 was composed of samples with relatively high contributions of PAH compounds to the estimated HHRs (see Figure S1 for each cluster profile in more detail). The increased PAH content in the environment was evident in the fluvial systems of the broader Moravian Silesian region (the Odra and Bečva Rivers). In this region, the anthropogenic pollution sources (coal processing, metallurgy) have generated higher PAH air concentrations [34] and greater contamination of agricultural soils [35]. The PAH contamination in floodplains of the Bečva River may be significantly influenced by the coal tar refinery (Deza Corporation) producing aromatic hydrocarbons [36]. Elevated concentrations of PAHs were also recorded in the floodplains along the Jizera River near the industrial center of Mladá Boleslav (automobile manufacturing). Last but not least, traffic-related emissions increased exposure to PAHs at several locations with a lower distance to highways. PAHs have been determined to be elevated in soils affected by highway traffic [37,38] and have been proved to significantly contribute to AhR-mediated activity and adverse health effects of traffic-associated pollution [39,40]. Ollivon et al. [41] described how car traffic and atmospheric washout might impact river contamination in the highly urbanized area within the Seine catchment. Elevated PAH contributions, associated with above-average health risks, were surprisingly recorded in the headwaters of the Elbe and Morava Rivers. This could only be explained by an atmospheric transport of PAHs that may increase their concentrations in remote freshwater ecosystems with no other pollution sources [42]. Using a regression tree model, Kubošová et al. [43] confirmed the crucial role of industry and other primary anthropogenic sources in soil contamination by PAHs. Yet, they also identified precipitation as the main transfer route of atmospheric PAHs to soils. The resulting regional patterns of PAHs in their stochastic approach using regression trees [43] corresponded well to our results of health risk patterns in floodplain areas.

Concerning Cluster 1, two regional centers could be distinguished. One was probably associated with the former contamination of the Spolana chemical plant at the middle reach of the Elbe River. The chemical plant grounds were contaminated with dioxins, chlorinated aliphatic hydrocarbons, and OCPs [44,45]. Studying the POP bioaccumulation in the Elbe River, Randák et al. [46] also found the highest concentrations of PCBs in chub muscle in fish from the Neratovice site. Thus, multiple sources may interact, resulting in a more complex soil pollution profile and corresponding health risks. The second regional occurrence of Cluster 1 was observed at the middle reach of the Morava River, where higher concentrations of organochlorine pesticides (especially HCHs and PeCB) were found. Studying POP dynamics in the dissolved phase in the water column of the Morava River and its tributaries, Prokeš et al. [47] found high aqueous concentrations of organochlorine hydrocarbons (especially PCBs and HCHs).

Finally, the characteristic features of Cluster 3 included higher contributions of DDT and its metabolites to the estimated HHRs. DDT and its metabolites dominated most soil samples from the catchment of the Berounka River (Central Bohemia), from the lower reach of the Eger River, and from a wider region of the South Moravian catchments. The floodplains of these lowland regions belong to traditional areas of intensive agricultural production over a long period of time. The observed regional pattern of Cluster 1 showed exceptional agreement with the predicted concentrations for DDTs in a regression tree model [43] and with some field observations of sediment geochemistry [48].

Given the results of the total estimation of HHRs for all soil samples, potential regional hot spots of human health impacts may be recognized (note the yellow circles in Figure 2 and Figure 5 highlighting the samples with HI_HUMAN_ > 1.0). In fact, if we display these samples in a ternary plot (Figure 2) or in the results of fuzzy clustering (Figure 5), the predominance of PAH-induced human health risks is clearly evident. Some exceptions to this included the Elbe samples collected close to the confluence with the Eger River and samples taken near the Neratovice site, which both showed more complex pollution profiles (elevated contributions of PCBs, HCHs, DDTs, and PAHs).

## 5. Conclusions

An elevated probability of human health impacts of soil pollution may be expected at several sampling sites under exactly defined exposure conditions. When combining both the magnitude of estimated HHRs (noncarcinogenic and carcinogenic effects) and the compositional variation of pollution profiles, several localities with high contents of PAHs proved to have the highest estimated HHRs. Concerning the intensive agricultural utilization of floodplains, the exposure of farmers can be considerable when they are exposed to polluted soils with significantly increased concentrations of POPs (mainly c-PAHs) and over long working hours. Since other population groups, such as residents and bystanders, may also be exposed to these compounds due to environmental pollution, a high degree of attention should be dedicated to human risk assessments associated with nonoccupational exposure in floodplain areas. This may especially be the case for the North Moravian region, where the population generally suffers from higher residential exposure due to air pollution. This study only focused on certain POPs and exposure scenarios and did not consider additional routes of exposure (dietary intake) or special vulnerable populations with specific exposure scenarios [49]. However, in agricultural areas it must be considered that concentrations of additional contaminants (especially formerly used or emerging pesticides [50,51], polychlorinated dibenzo-p-dioxins and dibenzofurans [52], heavy metals [3,53], or other micropollutants [54,55]) may also contribute to a higher probability of adverse effects. Likewise, health risks may be underestimated owing to the nature of the simple additive model avoiding all compound interactions and their synergic effects. According to the intensive farming in the floodplains and potential pathways for POP transfer to the human food chain, dietary exposure is expected to significantly contribute to HHRs. Various studies have provided strong evidence that the flooding of pastureland can indeed result in elevated concentrations of POPs in milk and meat produced on flood-prone land [52,56,57]. POPs are adsorbed on soil organic matter or in the plant rhizosphere so strongly that they are marginally transported to plant tissues [58]. Some problems might arise from the growing of feed crops for direct consumption, especially for plants producing consumable parts in the soil [59,60,61], since Trapp [58] presented a comparable adsorption of lipophilic organic substances within the rootzone with their adsorption to soil organic carbon. These risks may come into question for the localities in Cluster 3, where a high contribution of DDTs and metabolites to HHRs meets areas of intensive vegetable farming in floodplains near the Elbe and Eger confluence or in South Moravia. The regional patterns obtained in our floodplain study corresponded well with results of model-based predictions of POP concentrations in soils [43] and with some field observations of sediment, water, and biota [46,47,48]. This indicates the general role of floodplains as an effective sink of overall regional pollution. Since microbial degradation is a potential mechanism for the removal or transformation of POPs [62] and soil microbial characteristics have been proven to be very dynamic in human-altered riverine landscapes [63], special attention should be paid to the bioavailability mechanisms of POPs in these soils in the future. Moreover, filling the data gaps in understanding contaminant bioaccessibility is advisable to accurately assess human exposure to POPs via direct pathways [64].

Since a recent evolution of riverine landscape tends toward higher flood control in urban areas and flood-retarding inundation out of populated areas, agricultural floodplain soils will suffer the impacts of high water events. Hence, the results of this study were implemented in a national certified guideline for a complex assessment of soil pollution in flood-prone areas [65]. Additionally, these results were used to verify the relevancy of the human health limits recently adopted within the national soil protection legislation [30]. Purely from a methodical point of view, an exploratory analysis of the results of human health assessments using a compositional approach might be worth modeling and interpreting, especially when a clear dominance of c-PAH contributions outweighs the contributions of most other elements.

## Figures and Tables

**Table 1:** Exposure parameters for noncarcinogenic risk calculation.

Symbol	Parameter (Unit)	Value
THQ	Target hazard quotient	1
BW_c_	Body weight, child (kg)	15
AT_n_	Averaging time, noncarcinogens (days)	ED × 365
EF_r_	Exposure frequency, resident (day year^−1^)	250 (8 h/day)
ED_c_	Exposure duration, child (years)	25
IRS_c_	Soil ingestion rate, child (mg day^−1^)	100
RfD_o_	Oral reference dose (mg kg^−1^ day^−1^)	Chemical-specific
SA	Dermal surface area, child (cm^2^ day^−1^)	3470
AF	Soil adherence factor, child (mg cm^−2^)	0.12
ABS	Skin absorption factor (unitless)	Chemical-specific
IRA_c_	Inhalation rate, child (m^3^ day^−1^)	20
RfD_i_	Inhalation reference dose (mg kg^−1^ day^−1^)	Chemical-specific
VF_s_	Volatilization factor for soil (m^3^ kg^−1^)	Chemical-specific
PEF	Particulate emission factor (m^3^ kg^−1^)	Chemical-specific

**Table 2:** Exposure parameters for carcinogenic risk calculation.

Symbol	Parameter (Unit)	Value
SSL_i_	Contaminant concentration (mg kg^−1^)	Chemical-specific
TR	Target cancer risk	1 × 10^−6^
AT_c_	Averaging time, carcinogens (days)	25,550
EF_r_	Exposure frequency, resident (day year^−1^)	250 (8 h/day)
IFS_adj_	Age-adjusted soil ingestion factor ((mg year^−1^)/(kg day))^−1^	100
CSF_o_	Oral cancer slope factor (mg kg^-1^ day^−1^)	Chemical-specific
SFS_adj_	Age-adjusted dermal factor ((mg year^−1^)/(kg day^−1^))	361
ABS	Skin absorption factor (unitless)	Chemical-specific
InhF_adj_	Age-adjusted inhalation factor ((m^3^ year^−1^)/(kg day^−1^))	11
CSF_i_	Inhalation cancer slope factor (mg kg day)^−1^	Chemical-specific
VF_s_	Volatilization factor for soil (m^3^ kg^−1^)	Chemical-specific
PEF	Particulate emission factor (m^3^ kg^−1^)	Chemical-specific

**Table 3:** Summary statistics for the chemical concentrations of persistent organic pollutants (POPs) in the floodplain soil dataset (minimum: MIN; median: MED; maximum: MAX; spread expressed as median absolute deviation (MAD) and percentage of observations below the detection limit (% <DL)) and a summary of the hazard quotient calculation (median (MED_HQ) and its relative proportion to the median of the hazard index (%_HI)).

POPs		Chemical Measurement					Risk Estimation	
Compound	Abbreviation	MIN	MED	MAX	MAD	% <DL	MED_HQ	%_HI
		μg/kg				%	-	%
PCB 28	PCB28	0.06	0.10	1.37	0.02	38	0.0001	0.05
PCB 52	PCB52	0.05	0.10	0.56	0.00	58	0.0001	0.05
PCB 101	PCB101	0.03	0.13	1.18	0.05	37	0.00014	0.07
PCB 118	PCB118	0.02	0.10	0.34	0.07	57	0.0001	0.05
PCB 153	PCB153	0.08	0.32	4.66	0.25	6	0.000318	0.15
PCB 138	PCB138	0.06	0.32	3.36	0.25	15	0.00032	0.15
PCB 180	PCB180	0.05	0.28	4.68	0.24	9	0.00028	0.13
Pentachlorobenzene	PeCB	0.01	0.12	1.82	0.09	16	1.79 × 10^−7^	0.00
Hexachlorobenzene	HCB	0.13	1.21	8.66	0.95	0	0.000843	0.39
alpha-Hexachlorocyclohexane	aHCH	0.12	0.44	9.52	0.41	16	0.001189	0.56
beta-Hexachlorocyclohexane	bHCH	0.03	0.10	11.0	0.00	74	7.69 × 10^−5^	0.04
gamma-Hexachlorocyclohexane	gHCH	0.10	0.41	3.88	0.19	15	0.000164	0.08
o,p’-DDE	opDDE	0.01	0.10	35.4	0.02	41	1.47 × 10^−5^	0.01
p,p’-DDE	ppDDE	0.34	3.85	1923	4.05	0	0.000551	0.26
o,p’-DDD	opDDD	0.01	0.13	12.3	0.07	29	1.35 × 10^−5^	0.01
p,p’-DDD	ppDDD	0.04	0.52	38	0.52	8	5.42 × 10^−5^	0.03
o,p’-DDT	opDDT	0.05	0.49	329	0.55	9	5.58 × 10^−5^	0.03
p,p’-DDT	ppDDT	0.12	4.28	1082	4.77	0	0.000493	0.23
Naphthalene	N	4.38	10.4	648	5.09	0	0.000614	0.29
Acenapthene	Ace	0.06	1.67	589	1.39	1	3.73 × 10^−8^	0.00
Fluorene	Fluor	0.92	2.86	477	1.74	0	9.58 × 10^−8^	0.00
Anthracene	Ant	0.67	5.27	791	5.58	0	2.2 × 10^−8^	0.00
Fluoranthene	Fl	10.58	89.31	4268	95.2	0	2.95 × 10^−6^	0.00
Pyrene	Pyr	8.31	71.93	2966	75.5	0	3.11 × 10^−6^	0.00
*Benz(a)anthracene*	BaA	3.00	35.84	16,705	36.4	0	0.012255	5.73
Chrysene	Chr	4.58	45.81	1368	45.5	0	0.000158	0.07
*Benzo(b)fluoranthene*	BbF	5.58	53.30	1818	53.7	0	0.018431	8.62
Benzo(k)fluoranthene	BkF	2.08	23.05	624	22.3	0	0.000792	0.37
*Benzo(a)pyrene*	BaP	3.62	44.50	1475	43.7	1	0.152897	71.53
Indeno(1,2,3-cd)pyrene	Ipyr	2.77	33.05	966	31.3	0	0.011278	5.28
Dibenz(ah)anthracene	DBahAnt	0.10	3.29	58.43	3.37	1	0.011179	5.23

## Supplementary Materials

The following are available online at http://www.mdpi.com/1660-4601/15/6/1146/s1, Figure S1: Visualization of cluster profiles: relative weights of POP components for every cluster in the robust cluster analysis (isometric log-ratio (ilr)-transformed and centered data).

## Abbreviations

FCM	fuzzy c-means algorithm
HCHs	hexachlorocyclohexane isomers
HCB	hexachlorobenzene
DDTs	DDT isomers and their metabolites
HHRs	human health risks; OCPs: organochlorine pesticides
PAHs	polycyclic aromatic hydrocarbons
c-PAHs	carcinogenic polycyclic aromatic hydrocarbons
PC	principal component
PCA	principal component analysis
PCBs	polychlorinated biphenyls
PeCB	pentachlorobenzene
POPs	persistent organic pollutants
SSL	soil screening level
