# Supplementary material for: Which Compounds Contribute Most to Elevated Soil Pollution and the Corresponding Health Risks in Floodplains in the Headwater Areas of the Central European Watershed?
Source: Int J Environ Res Public Health. 2018 Jun 1;15(6):1146. doi: 10.3390/ijerph15061146 (PMC6025328; doi:10.3390/ijerph15061146)
Supplement: Supplementary file 1 [file ijerph-15-01146-s001.pdf]

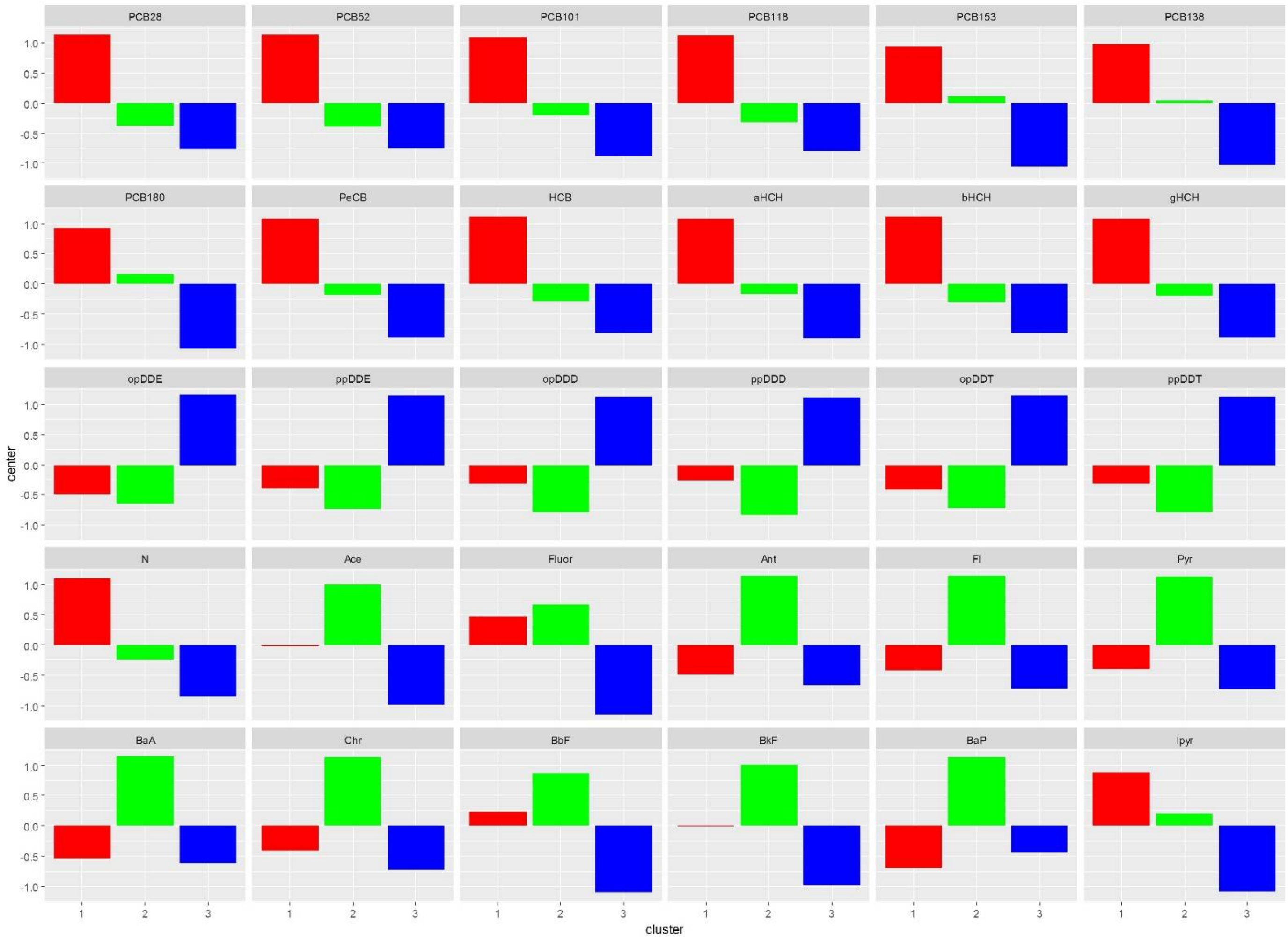

Figure S1. Visualization of cluster profiles: relative weights of POP components for every cluster in the robust cluster analysis (isometric log-ratio (ilr)-transformed and centered data).
